# *NGS-Logistics*: federated analysis of NGS sequence variants across multiple locations

**DOI:** 10.1186/s13073-014-0071-9

**Published:** 2014-09-17

**Authors:** Amin Ardeshirdavani, Erika Souche, Luc Dehaspe, Jeroen Van Houdt, Joris Robert Vermeesch, Yves Moreau

**Affiliations:** KU Leuven, Department of Electrical Engineering (ESAT), STADIUS Center for Dynamical Systems, Signal Processing and Data Analytics, Kasteelpark Arenberg 10, Box 2446, 3001 Leuven, Belgium; iMinds Medical IT Department, Kasteelpark Arenberg 10, Box 2446, 3001 Leuven, Belgium; KU Leuven, Center of Human Genetics Gasthuisberg, O&N I Herestraat 49, Box 602, 3000 Leuven, Belgium

## Abstract

As many personal genomes are being sequenced, collaborative analysis of those genomes has become essential. However, analysis of personal genomic data raises important privacy and confidentiality issues. We propose a methodology for federated analysis of sequence variants from personal genomes. Specific base-pair positions and/or regions are queried for samples to which the user has access but also for the whole population. The statistics results do not breach data confidentiality but allow further exploration of the data; researchers can negotiate access to relevant samples through pseudonymous identifiers. This approach minimizes the impact on data confidentiality while enabling powerful data analysis by gaining access to important rare samples. Our methodology is implemented in an open source tool called *NGS-Logistics*, freely available at https://ngsl.esat.kuleuven.be.

## Background

Next-generation sequencing (NGS) is a key tool in genomics, in particular to study inherited and acquired human genetic disorders [1]. Multiple projects now aim at mapping the human genetic variation on a large scale, such as the 1,000 Genomes Project [2], the UK 100 k Genome Project [3], or the Genome of the Netherlands project [4]. Meanwhile, with the dramatic decrease in price and turnaround time, large amounts of human sequencing data have been generated over the past decade [5]. As of August 2014, about 2,555 sequencers were spread over 920 centers across the world [6]. As a result, about 100,000 human exomes have been sequenced so far [7].

Crucially, the speed at which NGS data are produced greatly surpasses Moore’s law [5] and challenges our ability to conveniently store, exchange, and analyze the data. Data processing is needed to extract reliable information from sequencing data and it can be divided into two major steps: primary analysis (image analysis and base calling) and secondary analysis. When looking for variation in the human genome, secondary analysis consists of aligning/mapping the reads against the reference genome and scanning the alignment for variation. Both raw data (that is, the reads produced by the sequencer as well as their quality values, generally FASTQ files) and mapped reads (usually BAM files [8]) are large files occupying significant disk storage space. The collection of files resulting from the analysis of a single whole genome study can take up to 50 Gb of disk space [9]. This raises significant issues in terms of computing and data storage and transfer, with off-site data transfer currently being a key bottleneck.

Moreover, the analysis of NGS data also raises the major challenge of how to reconcile federated analysis of personal genomic data and confidentiality of data to protect privacy. In many situations, the analysis of data from a single study alone will be much less powerful than if it can be correlated with other studies. In particular, when investigating a mutation of interest, it is extremely useful to obtain data about other patients or controls sharing similar mutations. However, personal genome data (whole genome, exome, transcriptome data, and so on) are sensitive personal data. Confidentiality of these data must be guaranteed at all times and only duly authorized researchers should access such personal data. Moreover, important issues around informed consent also arise. What are acceptable uses of personal genomic data, in particular for data that have been collected primarily for a clinical purpose rather than for a research study? These questions are particularly hard because the legal landscape surrounding them and the interpretation of existing regulation varies from jurisdiction to jurisdiction, and is quickly evolving over time. We cannot resolve those questions here. What we do in this article is show what can be done from a technical perspective to balance data confidentiality and powerful data analysis for the benefit of patients and the community.

### Data sharing

Biology and especially genomics is a data-rich environment. Tools and methods are constantly being improved; each method having different features compared with previous versions. Since the output format of NGS sequencers has converged to standard formats over the past years, this provides a great opportunity for bioinformaticians to reconstruct their pipeline based on new methods and software to get better results. The reusability of data makes researchers eager to access each other’s data, but creates the additional challenge of providing this access at a sufficiently detailed level. We therefore aim at a solution that allows querying the data down to the level of aligned reads (BAM/SAM files) rather than only at the level of called variants (Variant Call Format (VCF) files) because those may vary significantly as calling algorithms evolve.

In addition, the number of similar cases needed to confirm a clinical or biological hypothesis has always been an issue. Gathering similar observations can lead to more accurate results and conclusions. In most situations, a change in one single nucleotide can affect the whole mechanism of a gene and consequently corrupt the behavior of the pathways involved, which could cause a disorder [10]. Therefore, comparison across multiple patients and controls is a key feature in studying single-nucleotide variants (SNVs) and in genomics research in general. In practice, smaller data sets and data from routine clinical work are often not shared, although they also have the potential to add information that may be useful for the analysis of other research projects. Another example is that of building and sharing a database of variant frequencies in unaffected individuals, which will help in excluding common variants from further analysis. This is especially important since common variants are highly variable between populations and thus regional databases will play a key role in interpreting variants for research and clinical purposes. Thus, some form of data sharing is highly desirable.

### Privacy

The current European legal framework [11] on data privacy is the main influence on our work, but similar issues arise in the US legal framework and elsewhere. Moreover, in a setting of international collaboration, any solution will have to fulfill all legal requirements and thus focusing on the slightly more conservative EU framework may be advantageous.

A discussion of privacy legislation is beyond the scope of this article, but a brief overview of some key concepts is useful to understand the motivation for our work.

Personal data [12] comprises any information relating to a person who can be identified, directly or indirectly, in particular by reference to an identification number or to one or more factors specific to his physical, physiological, mental, economic, cultural or social identity. It consists of any data for which someone can link the information to the person it originated from, even if the person holding the data cannot make this link.

Personal data can only be processed under the three key principles of transparency, legitimacy, and proportionality. Transparency implies that individuals have a right to be informed when and how their personal data are being processed. This includes the need for appropriate consent for processing of the data, and the need for identifying all people processing the data. Legitimacy implies that personal data can only be processed for specified explicit and legitimate purposes and may not be processed further in a way incompatible with those purposes. Proportionality implies that personal data may be processed only insofar as it is adequate, relevant and not excessive in relation to the purposes for which they are collected or further processed. This includes the right to correct or withdraw information. By contrast, and importantly, data that are not personal - which means data that cannot be linked back to a specific individual - are not subject to these requirements.

Personal genomic data comprises any substantive amount of genomic sequence data (for example, human whole genome, exome, transcriptome, or epigenomic sequencing) that are deemed personal by way of being unique to each person and fixed. Importantly, this implies that removing classical identifiers from the data (names, social security identifiers, patient identifiers, or pseudonymized versions of those) is insufficient to keep the data from being personal.

### Data management

Data control is an important and complex issue when dealing with personal data. It is therefore necessary to determine and know ‘who has access to what’. This corresponds to the key principle of ‘transparency’ in the management of personal data. In most research institutes, it is the responsibility of the principal investigator (PI), the head of a group, to supervise the access of researchers to the data of a study, after approval by an institutional review board. In a simple scenario, the PI defines the study and assigns the researcher to it, and only those researchers need to access the data. However, there is often a need or desire to share data with other researchers from the team, and it can also often be useful to share the data and results with other groups within or outside the institution. Privacy principles do not require that the number of people accessing the data be minimized at all cost, but rather that the process be managed transparently, that the data be used for legitimate purposes, and that only people with a legitimate interest access the data.

A potential solution to manage access to personal genomic data is the use of access control lists (ACLs). An ACL can be defined and assigned to a corresponding study, and researchers can then be assigned to this ACL. This feature allows control of which researcher has access to which study. Many operating systems support ACLs, although this is not yet a fully standard feature. For a center with several groups and several studies, however, it can quickly become difficult to maintain all these access permissions as the number of samples and studies increases. There is therefore a need for a data management system that can handle these permissions efficiently.

### Data storage and processing

Monitoring of pipelines shows that there are several bottlenecks in terms of data storage and processing. Processor speed, virtual memory, and disk storage are the most frequently observed bottlenecks. To analyze large amounts of data, organizations will increase their computational power by upgrading their existing servers (scale up) and nowadays by adding computing and storage servers (scale out) to their hardware facilities [13]. Therefore, a well-designed infrastructure supported by powerful computational resources and organized storage servers is essential. As mentioned before, by having access to similar cases and by comparing different studies, researchers can make more accurate conclusions. Consequently, having access to other data across studies is necessary. However, it is often far from optimal to store copies of additional data sets next to the user’s own data because of the significant costs involved, as well as the time necessary to obtain the data and to transfer it locally. Being able to investigate other data sets without actually moving the data could increase research efficiency.

### Aims

Our ultimate goal is to provide a way for biomedical researchers to ask high-level questions, such as ‘what is the frequency of appearance of a single mutation across the large population’ or ‘how many patients with disease D have a mutation in gene G?’ transparently. Our system also allows detailed anonymized analysis of genome sequencing data at single positions in the genome, for example, to distinguish rare mutations from highly variable or poorly sequenced positions in the genome. To address all the issues described above, we have developed a data infrastructure, *NGS-Logistics*, that fulfills all requirements of a successful application that can process data inclusively and comprehensively from multiple sources while guaranteeing privacy and security by avoiding the exchange of raw data. *NGS-Logistics* is a web-based application providing a data structure to analyze NGS data in a distributed way. The data can be located in any data center, anywhere in the world. *NGS-Logistics* provides an environment in which researchers do not need to worry about the physical location of the data. With respect to users rights, queries will be sent to each remote server. The host will process the request and return the results back to the main server where all the privacy limitations are controlled for the data. Once the results are ready, the end user can see the desired information. In the next section, we provide a detailed description of functionalities of the system and its implementation.

## Implementation

The *NGS-Logistics* application package consists of three modules: 1) administration; 2) query manager; 3) primary user interface.

**Figure 1:**
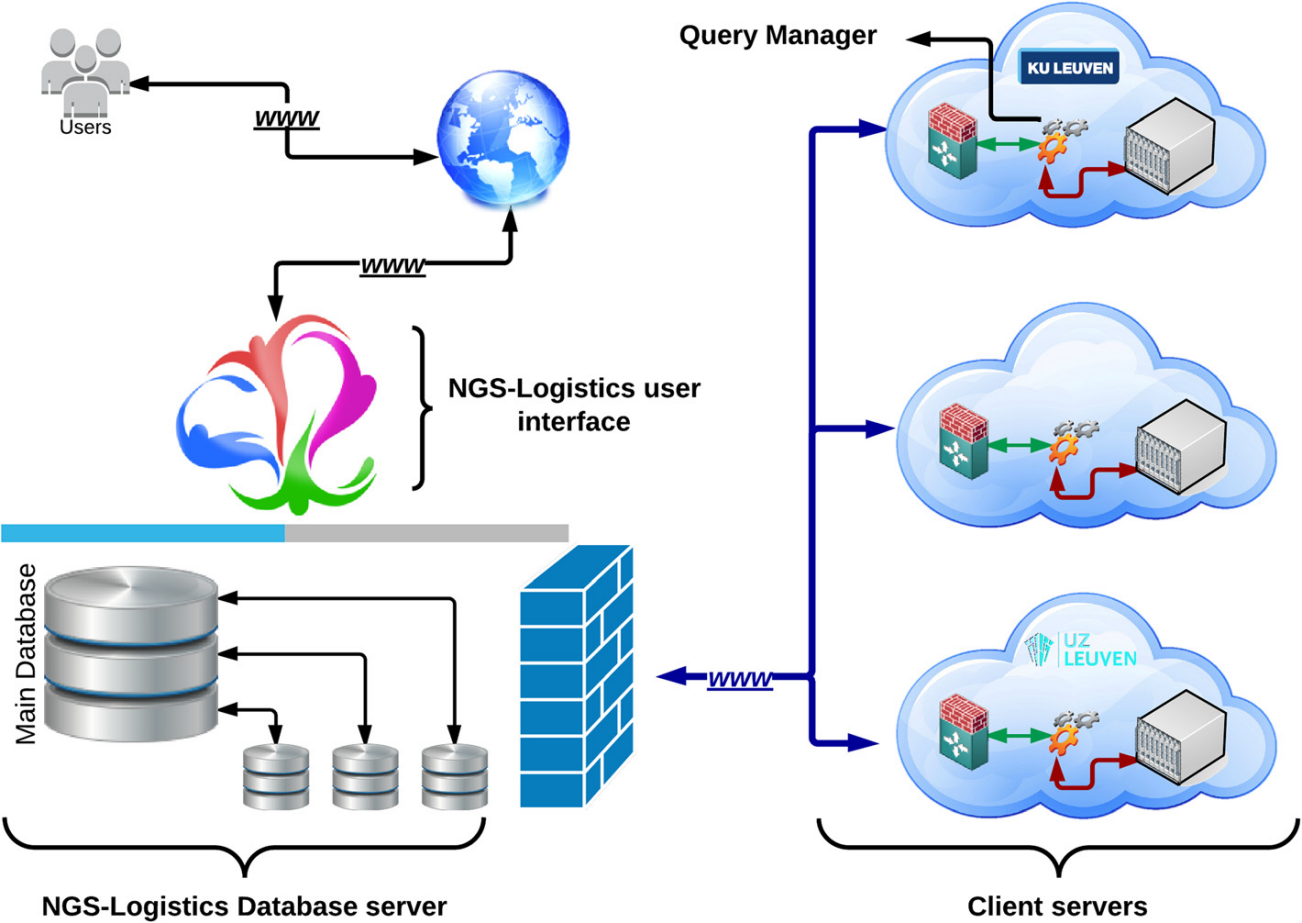
***NGS-Logistics*** **components.** Users pass their queries from the *NGS-Logistics* web interface to the clients. Requests are stored and scheduled in the main database. Each center has one database, being the only way of communication between centers and the main system. Centers and their databases are connected through a secured connection, to which only valid and trusted IPs are allowed to connect. The query manager is responsible for tracking and running the request, as well as collecting and returning the results to the main system.

Figure 1 shows the components of *NGS-Logistics. NGS-Logistics* is designed as a multi-tier architecture that allows the development of a flexible and reusable application. It consists of three tiers: presentation, application processing, and data management. Different software packages are used depending on the nature of the component. Currently, Java is used for the administration and query manager modules, while ASP.NET is used for our user interface. Microsoft SQL Server is used as our database management system. These are local design choices and the application could be redeployed fairly easily using other programming technologies. Each one of these components and their characteristics is further explained below.

### Administration

**Figure 2:**
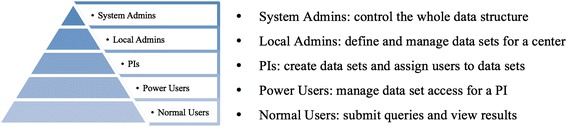
***NGS-Logistics*** **user types and their access levels.**

The administration module mainly focuses on controlling the access rights of the users. It is the place where the administrator sets up access control among users, so that confidentiality of personal genomic data can be guaranteed. The system administrator can also control the type and volume of queries that the different users are allowed to submit. The user interface is designed in such a way that the complexity of users’ access controls is hidden. For finer control over users, users can be assigned roles at different levels (Figure 2).

The system administrator defines the centers and their local admins. Local admins are responsible for ensuring that the sample list is kept updated and the samples are assigned to the authorized PIs. They also manage the users’ accounts, assign samples to data sets, and allocate data sets to users or groups of users. Data sets can be private or public (that is, accessible to all the users of the system) depending on the characteristics of the samples that they contain.

#### Sample list

The sample list is the list of all files that each center makes available through *NGS-Logistics*. The sample list can be updated either automatically or manually by the local admin. Several fields such as the ‘Sample Local Name’ , ‘PI Name’ , ‘Sample Type’ , and ‘Sample Reference version’ are compulsory for each sample. At the time of insertion, *NGS-Logistics* automatically assigns an internal Sample ID to each sample to pseudonymize the actual name (Sample Local Name) given to the sample by the owner from the rest of the users. (Note that pseudonymization does not stop the data from being personal because the genome or exome sequence is unique to each individual). Then it uses the ‘PI Name’ and ‘Sample Type’ fields for recognition of the type of sample (Research or Diagnostics) and assigns them to the owner of the sample (PI Name). The ‘Sample Reference Version’ shows the version of the human genome build to which the sample has been aligned. Since samples can be aligned to different builds of the human genome, the system will use this information to aggregate query results. To do so, we use the UCSC LiftOver database [14].

### Query manager

The query manager is a component that picks up the request at each center, runs the task, gathers the results, and returns them back to the main system. Since there are different infrastructures and software packages in different centers, we have tried to keep the query manager as simple as possible. To establish a connection between the server and clients, we only need to configure the query manager by changing a small list of parameters, such as the address of the executable applications and of the data (BAM files). As we need to integrate all results from different sources, we query BAM files with GATK [15]. The output looks like a standard VCF file [16], which is exported from the different centers to the main system. Standard GATK variant caller output has the following fields: Chromosome, Position, dbSNP ID, Ref, Alt, Qual, Filter, Info Format, and Format Values.

### User interface

The user interface plays an important role in the success of an application. Well-designed user-friendly interfaces have a great impact on the users’ activities. Therefore, we have tried to make the interface as simple and intuitive as possible. The *NGS-Logistics* interface allows the users to submit their queries, track them, and visualize the results. To further explain this, we divide the interface into three categories: user settings, query builder, and results.

#### User settings

User settings allow users to alter their information, create their own data sets, assign samples (to which they have authorized access) to these personal data sets, and activate their data sets. Users can choose the data set they want to investigate from the list of those to which they have authorized access. This procedure is called Data Set Activation. Activating a data set is important: if no data set is activated, no detailed results will be available (in the results section, we will discuss what kinds of detailed information can be investigated). This feature is part of our security system and privacy agreement. In this way, we guarantee that users can only see information they are authorized to see. Besides detailed information, some summary values (such as the total number of appearance of a variant or average coverage) are displayed.

Users can also ask for access to samples and data sets from other groups. In this case, the owner of the data must review the request and grant access to the data or not.

#### Query builder

Two types of queries are available: point and area statistics. GATK is used to call variants for both types of queries but results and statistics are shown slightly differently for each type of query. Since GATK provides two variant callers, users can choose between HaplotypeCaller and UnifiedGenotyper. In point queries, users must fill in the chromosome and position and have the possibility to genotype reference sites (allSitePLs); whereas in area queries, they have to provide the chromosome number and start and end positions, or a gene name. After submitting the query, a unique request ID is assigned to the request. In the background, the system automatically sends the query to the centers and waits for results. Meanwhile, through the request tracking option, users can see the status of the request. Since the process really depends on the facilities and workload of each center, we cannot estimate the duration of each process but display the progress by center.

## Results and discussion

All functionalities of *NGS-Logistics* will be illustrated by querying one gene. Another example will demonstrate how *NGS-Logistics* can help in interpreting variants. A demonstration run was performed and successfully analyzed samples located at two centers (KU Leuven and UZ Leuven) with approximately 1,500 exome samples in total.

### Use case one

**Figure 3:**
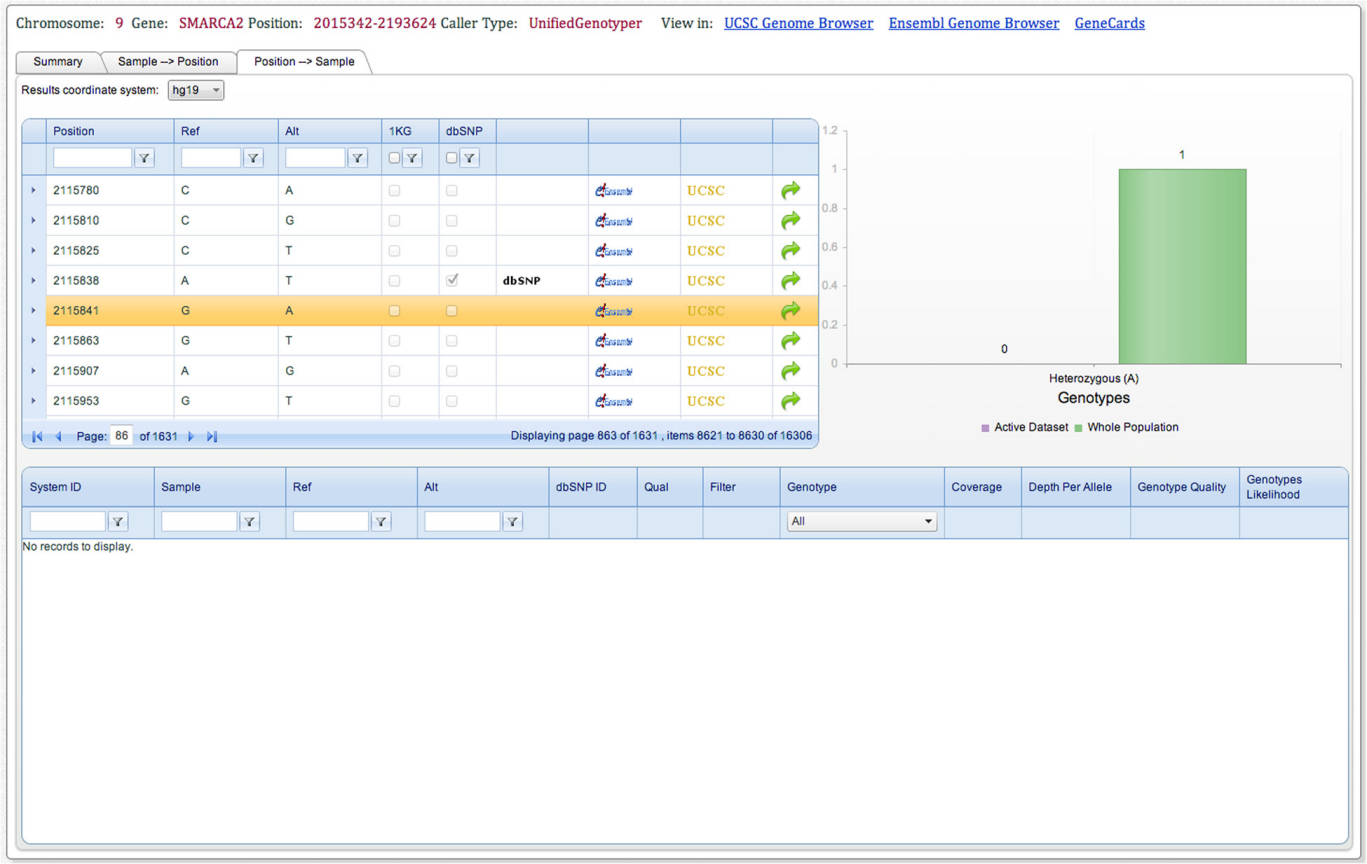
***NGS-Logistics*** **area query results page (Position to Sample section) for** ***SMARCA2*** **.** Results demonstrate that at the selected position (chr9:2115841, build 19) a G to A mutation can be observed with heterozygous genotypes. The graph shows the differences between the active data set and the whole population. Users can see the list of samples to which they have access and related information in the detail table (if any is available). For further inquiries about the selected position, links to the public databases are provided. Also users are able to submit a single point query if they are interested to see more details about the selected position.

We use the example of *SMARCA2*, a gene located on chromosome 9 whose heterozygous mutation causes Nicolaides-Baraitser syndrome [17]. We start with the area query. An area can be queried using a gene name or a region (defined by chromosome number and start and end positions). The area query results section is divided into three sections: ‘Summary’ , ‘Sample to Position’ , and ‘Position to Sample’ . The top part of each section describes the query and includes, if possible, links to well-known online databases, such as the UCSC and Ensembl genome browsers. The ‘Summary’ section displays the total number of samples queried in each center and the total number of samples to which they have access. Results can be investigated by checking the list of available mutations per sample (‘Sample to Position’ section) or by checking the list of all mutations for the selected region, how frequently they occur, and which genotypes are observed in all samples from all centers (‘Position to Sample’ section) (Figure 3). We refer to all samples from all centers as the ‘Whole Population’. Note that the ‘Whole Population’ contains the samples from the active data set. One of the SNVs reported in *SMARCA2* has the dbSNP id *rs281875187* (chr9:2115841, build 19). As shown in Figure 3, this SNV is not observed in any sample from the active data set, but in the whole population, one sample is heterozygous. If other samples were found with this mutation, it would be of direct interest to a researcher to contact the PI responsible for this sample, for example, to investigate whether or not this patient is affected by Nicolaides-Baraitser syndrome to confirm or weaken the hypothesis of association between Nicolaides-Baraitser syndrome and *SMARCA2* mutations, or to collect biological samples from patients for further biological studies.

**Figure 4:**
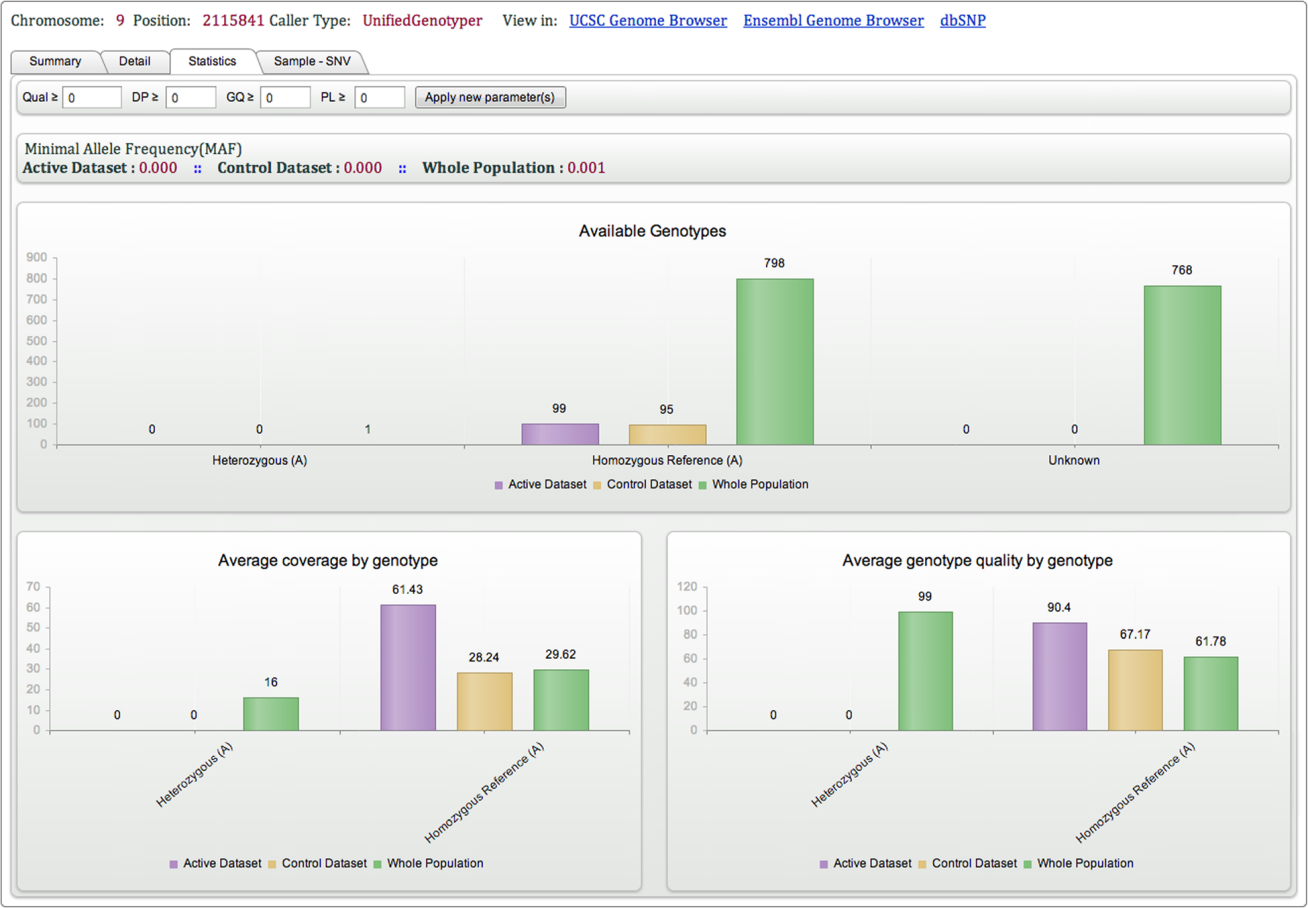
**Single point query result page (Statistics section) for chr9:2115841.** The query of chr9:2115841 shows that only one sample is polymorphic at this position. All samples that can be genotyped at this position from the active data set, the control data set and the whole population are homozygous reference. The MAF of this variant in each data set is thus very low.

**Figure 5:**
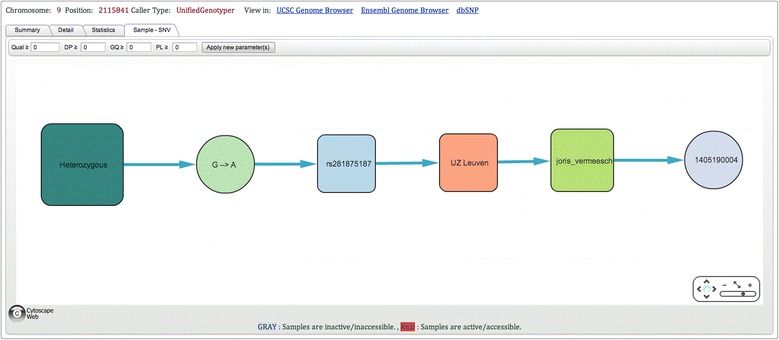
**Single point query results page (Sample to SNV section) for chr9:2115841.** Those samples that have a mutation are clustered based on their genotypes, dbSNP ID, Center, and Sample Owner name. Samples are labeled by their System Sample ID, which is different from the actual sample name, and are color-coded according to the user’s active data set (red = active/accessible, grey = inactive/inaccessible).

The single point query allows users to investigate a single position. The single point query results page is divided into four sections: ‘Summary’  , ‘Detail’ , ‘Statistics’ , and ‘Sample to SNV’. Similarly to the area query, the top part of each section contains information related to the query, as well as links to dbSNP and the UCSC and Ensembl genome browsers. The ‘Summary’ section displays information on the total number of samples queried in each center and summary results (number of samples genotyped as the same as or different to the homozygous reference, minimum, maximum and average quality scores). The ‘Detail’ section contains the GATK output for the samples of the active data set. Results are available for download. The ‘Statistics’ section provides an overview of the different genotypes and their quality values. Graphs are divided into three sets of data: the active data set, the control data set and the whole population (Figure 4). The control data set includes unaffected, unrelated individuals. Usually these individuals are either unaffected parents or unaffected siblings of affected patients. As such they might carry one pathogenic mutation. Each center can provide a list of these individuals. The minimal allele frequency (MAF) is calculated for each data set. A call is assigned to each sample and by default no quality filtering is performed by *NGS-Logistics*. However, users are free to select good quality variants according to quality, depth, genotype quality and/or PL (phred-scaled genotype likelihood) values. Finally, the section ‘Sample to SNV’ is the demonstration of the relation between available genotypes and samples (Figure 5). Samples are clustered based on their genotype, dbSNP ID, center, and PI name. Every sample is assigned a pseudonymized System Sample ID, different from the real sample ID. This identifier can be used to request access to the sample details. Samples for which the user is not allowed to see the details are colored in grey. This system allows the quick identification of the person to contact to get access to a relevant new sample.

### Use case two

**Figure 6:**
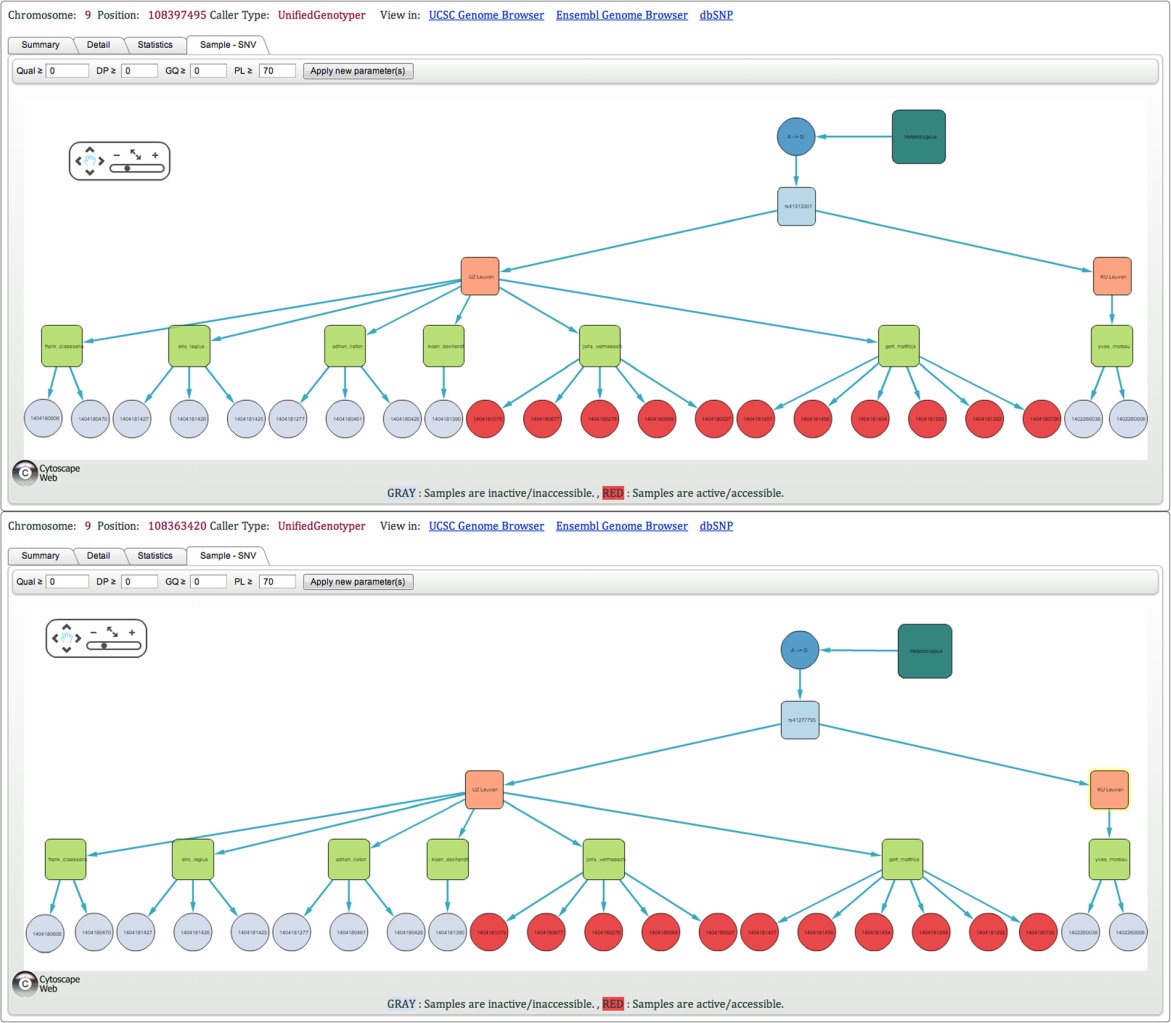
**Single point query results page (Sample to SNV section) for chr9:108363420 and chr9:108397495, build 19.** For both positions variants genotyped with PL <70 are filtered before counting polymorphic samples (PL: phred-scaled genotype likelihood). Since all 22 individuals carried both variants in a heterozygous state, we are able to conclude that the variants are on the same allele.

The variant analysis of three patients with a congenital disorder of glycosylation revealed two good quality heterozygous variants (chr9:108363420 and chr9:108397495, build 19) in the fukutin gene, responsible for muscular dystrophy-dystroglycanopathy. Since fukutin is a recessive gene disorder, two heterozygous variants on different alleles or one homozygous variant could cause the disease. The use of *NGS-Logistics* showed that both variants were reliably genotyped as heterozygous in 22 individuals and had a MAF of 0.05 and 0.03 in the control data set and the whole population, respectively. The ‘Sample to SNV’ graphs (Figure 6) show that the same 22 individuals carried both variants in a heterozygous state, indicating that the variants are on the same allele. Consequently, the combination of the two variants found in three of our patients cannot cause the disease.

## Conclusions

There is currently no comprehensive solution for personal genomic data sharing. The Database of Genotypes and Phenotypes (dbGaP) [18] and the European Genome-phenome Archive (EGA) allow the exchange of personal genomic data. However, some important limitations remain. The process of securing approval for data access is time consuming and involves significant administrative overhead because the process involves legal contracting between the requesting institution, dbGaP or EGA, and the institution providing the data. dbGaP or EGA acts as broker between institutions and maintains the data sets under escrow until approval of the requests. Data transfer to the receiving party, reprocessing of the data, and maintenance of the data locally represent significant hurdles for non-specialists and require significant computational resources. The need for reporting and project closure adds further to the administrative overhead. As a result, there is a major barrier to using those resources and the available data are not being exploited to the full extent. It also slows down the adoption of those systems by institutions that could be providing significantly more data than is happening in practice.

Another solution for data sharing is to rely on vendor solutions, such as Illumina BaseSpace, or more recently Google Genomics. Well-integrated vendor solutions are tempting because they could allow easy access to data sets from collaborators spread across the world through cloud-based solutions. However, it is unclear how such solutions will support a privacy-enhancing framework beyond leaving access decisions entirely at the responsibility of individual researchers. Moreover, such solutions could present a significant risk of network effect. If significantly more and more data become available through a given vendor, this solution will acquire a major advantage over the competition. Given broadly similar features, the network with the most data is by far the most valuable because it allows more research questions to be answered. A dominant network could possibly wipeout all significant competition, and gain a permanent lock-in over significant amounts of personal genomic data worldwide. Therefore, issues of platform interoperability and data freedom are essential when considering vendor solutions for personal genomic data sharing.

Further access to the samples by providing a researcher with direct access to given samples after a request is subject to the standard process. Does the consent provided by the patient or guardian allow for legitimate access by the third party? Or is additional consent or approval by the institutional review board necessary? This process cannot be automated at this point because the answer depends on the specific details of the request and on the legal framework imposed by the jurisdiction or institution. However, the fact that all users and requests are tracked provides a high level of transparency, which should ease this process greatly.

A key design choice in *NGS-Logistics* has been to provide data analysis at the level of aligned reads (BAM files) rather than called variants (VCF files). While deploying a system handling only VCF files might be easier, we believe that for detailed analysis of a single nucleotide position it is desirable to use a uniform assessment of variants across all samples, which will be difficult to guarantee for legacy VCF files produced at different times using different pipelines. Besides querying BAM files, *NGS-Logistics* allows the genotyping of reference calls, which are often not included in standard VCF files. Knowing whether a sample is homozygous reference at one position or whether the position is not covered is important since it allows a better assessment of the MAF.

One way in which the power of the system can be increased is by making healthy control population sets available through the system. Currently, the ‘Whole Population’ contains all samples available in the system regardless of clinical diagnosis. Once a relevant mutation has been identified, samples can be tracked down, and clinical information can be gained about these samples. However, the ‘Whole Population’ will contain samples that can be considered ‘cases’ as well as ‘controls’ without distinction. This prevents us from implementing any form of association scoring in *NGS-Logistics*. So far we have one control population but it be useful to add phenotypic information in the next phase, so that association scores can be calculated for mutations between the active data set and a control data set with respect to their phenotype. Promising mutations can then be evaluated in detail in the system. Similarly, another difficulty for any association analysis is that samples from the same patient could be processed at different sites (that is, seen by physicians at different clinical centers) with no communication that these samples are identical. This would skew any statistics of the presence of variants in the population. Using a hash reference derived from the genome of the patient, which would identify each patient uniquely and thus allow recognition of the fact that the same patient is present at two centers, would alleviate this problem. However, it is unclear how such a hash reference could be derived from noisy genome information and also such an approach goes against the idea of minimizing the amount of personal information shared because it provides a unique identifier for each patient. Our approach allows drilling down through the data to the lowest level and contacting PIs responsible for different patients and therefore eventually identifying that different records correspond to the same patient.

Finally, the main direction for further development of our system is that it currently does not support clinical information. Important technical issues make handling clinical information challenging. Clinical information is often managed and transferred separately from sequencing data. Researchers may often have sequencing data, but little clinical information beyond a few overly broad labels (for example, tumors versus controls), which makes query by others difficult. In practice, clinical information in genomic research projects is often managed in an unstandardized way without reliance on structured information, such as ontologies. Even if data are described in a structured way, there are currently only limited options to cover all clinical domains beyond Unified Medical Language System (UMLS) or Systematized NOmenclature of MEDicine Clinical Terms (SNOMED), which have important limitations. Extracting clinical information automatically from hospital information systems remains extremely challenging and will be done differently in each center. For these reasons, integrating clinical information into *NGS-Logistics* remained beyond the scope of this article. Developing a full clinical information management system is unrealistic and would be too cumbersome for users. A more realistic direction is to provide a simple way to annotate samples with either free text or, better, with Resource Description Framework (RDF) triples (or similar concepts) that allow annotation using properly referenced ontologies. Both functionalities should be present so as not to exclude users that can only provide free text annotation. Text search and SPARQL-like (SPARQL Protocol and RDF Query Language) querying would then allow identification of relevant samples based on clinical information. This will make it possible to carry out queries such as ‘which mutations are significantly more frequent in clinical population A versus clinical population B across multiple centers?’

The key concept in *NGS-Logistics* is to let users query SNVs across data sets in multiple centers. Users get a full description of the results for the samples to which they have authorized access, while getting relevant summary information for other samples. This summary information is sufficient to carry out key research tasks, such as identifying relevant mutations in a patient population. The footprint on personal genomic data from other patients is kept to a bare minimum. The frequency of mutation at a given position in the ‘Whole Population’ is not personal data and thus not subject to restrictions. The ability to identify a specific pseudonymized sample carrying a specific mutation provides *stricto sensu* access to personal genomic data. However, the amount of information is kept minimal (one single base-pair position at a time), the identifier is pseudonymized (to avoid any possibility of direct identification), and all users and requests are tracked by the system. The interest of broadly allowing *bona fide* researchers to identify relevant samples for further research or to support clinical diagnosis fulfills the legitimacy principle of data privacy. The fact that users and requests are tracked at all times contributes to the transparency principle. Finally, the fact that information about samples beyond the authorized ones is kept to non-personal summary statistics, except for a single backlink through a pseudonymous identifier, contributes to the proportionality principle. As such, we believe our system represents a best effort to enforce a high level of data protection while providing a powerful solution for clinical genomic research and diagnosis support.

## Availability and requirements

- **Project name**: *NGS-Logistics*
- **Project home page**: https://ngsl.esat.kuleuven.be
- **Operating system(s)**: Platform independent
- **Programming language**: ASP.Net and Java
- ***NGS-Logistics*has been tested on the following browsers**: Google Chrome, Mozilla Firefox and Safari

## Abbreviations

ACL: access control list
dbGaP: Database of Genotypes and Phenotypes
EGA: European Genome-phenome Archive
MAF: minimal allele frequency
NGS: next-generation sequencing
PI: principal investigator
RDF: Resource Description Framework
SNV: single-nucleotide variant
VCF: Variant Call Format
